# The epidemiology of Norovirus in the Middle East and North Africa (MENA) region: a systematic review

**DOI:** 10.1186/s12985-017-0877-3

**Published:** 2017-11-10

**Authors:** Khalil Kreidieh, Rana Charide, Ghassan Dbaibo, Nada M. Melhem

**Affiliations:** 1 Medical Laboratory Sciences Program, Faculty of Health Sciences, American University of Beirut, P.O. Box 11-0236, Riad El Solh, Beirut, Lebanon; 2 Department of Epidemiology and Population Health, Faculty of Health Sciences, American University of Beirut, Beirut, Lebanon; 3 Department of Pediatrics and Adolescent Medicine, Division of Pediatric Infectious Diseases, Faculty of Medicine, American University of Beirut, Beirut, Lebanon; 4 Center for Infectious Diseases Research, Faculty of Medicine, American University of Beirut, Beirut, Lebanon

**Keywords:** Norovirus, Acute gastroenteritis, Middle East and North Africa, Diagnosis, Seasonality

## Abstract

Norovirus (NoV) is considered the second leading cause of viral acute gastroenteritis (AGE). To our knowledge, there are no systematic reviews assessing the role of NoV in AGE in the Middle East and North Africa (MENA) region. Consequently, we conducted an extensive systematic literature review on articles studying NoV in the 24 countries of the MENA region during the past 15 years (2000-2015). The methods and reporting were set according to the 2015 PRISMA-P and based on the elements from the international prospective register of systematic reviews (PROSPERO). We retrieved 38 studies meeting our predefined inclusion criteria and were used to extract full data. Studies reporting on NoV were conducted in 15 out of the 24 countries of the region. The reported NoV infection rates in MENA countries ranged between 0.82% and 36.84%. The majority of studies were clinical observational studies assessing NoV rates mainly among children. Participants were recruited from in- and outpatient clinics. NoV infection was reported all year round with with peaks observed mainly during cold months. GII.4 was the predominant genotype detected in stool of participants as reported by 16 out of 25 studies (64%). Overall, there is an increasing recognition of NoV as an important causative agent of AGE across all age groups in the MENA region. Further studies are needed to assess the national and the regional burden of NoV among different age groups, its molecular diversity and seasonal variability.

## Background

Diarrheal disorders still mark the second highest burden among all communicable diseases [1] with an estimated 1.4 million yearly deaths and 89.5 million disability-adjusted life-years (DALYs) [1, 2]. Recently, noroviruses have been recognized as a leading cause of both sporadic and epidemic acute gastroenteritis (AGE) across all age groups seeking medical care in emergency departments, outpatient clinics and the community [3–6]. Norovirus (NoV), the second leading cause of viral AGE worldwide [7] after rotavirus, is responsible globally for substantial morbidity and mortality rates in both developed and developing countries [8–10]. It is estimated that 212, 000 deaths are caused by NoV yearly worldwide with 99% of these occurring in middle- and high mortality countries [11]. In the USA, NoV contributes to at least 20 million illnesses per year leading to 56,000-71,000 hospitalizations and 570-800 deaths [12]. In Europe, NoV was reported to be responsible for 5.7 million infections, 800,000 medical visits, 53,000 hospitalizations and 102 deaths among children less than 5 years between 2003 and 2013 [13]. Moreover, NoV is a major cause of gastroenteritis outbreaks accounting for at least 50% of the investigated cases [14]. NoV has been associated with diarrheal diseases among different age groups [15, 16]. In children less than 5 years old, the incidence of NoV is five times higher compared to other age groups [17] causing 18% of diarrheal diseases [18]. Global reports suggest that 70% of pediatric norovirus cases affect children in the 0-4 year age group [19] with greater infection rates in low-income countries and among inpatients.

Recently, the World Health Organization (WHO) positioned NoV in the global estimates of the burden of foodborne diseases [20] as the most common cause of morbidity and mortality and the fourth in DALYs’ burden [21]. These numbers reflected estimates for all age groups and modes of transmission and prompted researchers to report on the economic burden exerted by NoVs globally. NoV results in a global economic burden of $4.2 billion on health system costs with two-thirds affecting children less than 5 years old [22].

In recent systematic reviews assessing the role of NoV in AGE worldwide, the pooled prevalence of NoV was estimated at 18% [3] with winter seasonality [23]. The prevalence of NoV was reported to be higher in community settings (24%) followed by outpatient (20%) and inpatients settings (17%). Moreover, the introduction of rotavirus vaccine contributed to ranking NoV as the most common cause of gastroenteritis among children [24, 25]. Importantly, these reviews highlighted the lack of data from high mortality settings and low-income countries and the need for more studies to understand the role exerted by NoVs on the burden of diarrheal diseases. This is especially true since data on the regional distribution of NoV are only available from developed countries like USA, Europe and Japan [12, 17, 26–28]. Nineteen studies from fourteen African countries were recently reviewed by Mans et al. [29] whereby authors identified the lack of data on NoVs from diverse age groups in Africa in agreement with previous reports from low- and middle-income countries [3].

To our knowledge, there are no systematic reviews reporting on the NoV-associated AGE in the Middle East and North Africa (MENA) region. In this review, we present the reported frequencies of NoV-associated gastroenteritis in countries of the region in different age groups, the type of tests used to report NoV, the predominant circulating genotypes compared to globally reported ones as well as seasonality patterns in the region.

## Methods

### Search strategy and selection process

An extensive systematic literature search was carried out on articles studying NoV in the 24 countries of the MENA region during the past 15 years (2000-2015). These countries are: Algeria, Bahrain, Djibouti, Egypt, Iran, Iraq, Israel, Jordan, Kuwait, Lebanon, Libya, Mauritania, Morocco, Oman, Palestine, Qatar, Saudi Arabia, Somalia, Sudan, Syria, Tunisia, Turkey, UAE and Yemen.The methods and reporting of this systematic review were set according to the 2015 preferred reporting items for systematic review and meta-analysis protocols (PRISMA-P) [30] and based on the elements from the international prospective register of systematic reviews (PROSPERO) [31]. The search strategies were developed using the proper Medical Subject Headings (MeSH) terms and keywords related to NoV and the 24 countries listed above. To ensure the comprehensiveness and completeness of the search [32], six electronic bibliographic databases were used: Medline (OVID), PubMed, EMBASE, COCHRANE, SCOPUS and Web of Science. Moreover, in order to rule out any source of bias to the selected studies and in order to account for hard to reach material, unpublished literature in theses, dissertations and grey literature were screened through ProQuest and OpenGrey databases. The Global Health Library (GHL) was used to retrieve studies published on the regional databases namely: Index Medicus for the Eastern Mediterranean Region (IMEMR) and African Index Medicus (AIM).

All relevant studies were sent to the citation manager Endnote (version X7.1) where duplicates were removed. The titles and abstracts of the exported studies were extracted and screened by two independent reviewers for relevance according to the inclusion and exclusion criteria. Disagreements were resolved by consensus of a third reviewer.

We included the studies that reported the prevalence of NoVs in AGE in any of the 24 countries of the MENA region and published between 2000 and 2015. These studies should have reports on the total number of stool samples tested for NoV along with positive occurrences. Moreover, studies were included when the use of standardized laboratory techniques for the detection of the virus was clearly reported. These assays include enzyme immune assay (EIA), enzyme linked immunosorbent assays (ELISA), immunochromatography (IC), latex agglutination (LA), reverse transcriptase polymerase chain reaction (RT-PCR), real time RT-PCR (rRT-PCR), and sequencing.

We excluded review articles, case studies, clinical trials, animal and environmental studies. We also excluded studies that did not have English abstracts. Studies lacking the number of participants, the number of positive cases or the percentages that allowed these raw numbers to be calculated were also excluded.

## Data extraction

Based on the pre-defined inclusion and exclusion criteria, the full-texts of potentially eligible or relevant titles and abstracts were screened. Following screening and using a standardized data sheet, two reviewers extracted the data independently from eligible studies and assessed the inclusion criteria in details. The abstracted information was checked by a third reviewer. The following information was extracted from each study when available: country, population studied, number of cases tested, positive rate, diagnostic method used, predominant genogroups or genotypes and seasonal peaks. The population studied was recoded into participants’ setting, i.e. community, outpatient or inpatient. When studies did not report the positive rates of NoV infection, we calculated the former using the sample size and the number of positive cases.

## Results

### Study selection and characteristics

**Fig. 1:**
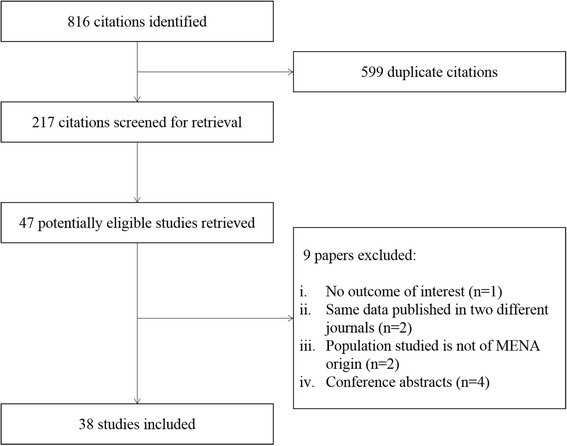
Flow diagram of NoV study selection in MENA countries, 2000-2015

Our literature search identified 816 articles on NoV in the MENA region. After removal of duplicates, we assessed the eligibility of 217 citations based on their titles and abstracts (Fig. 1). We retrieved 47 eligible articles out of which 9 were excluded based on our inclusion criteria. Consequently, 38 studies met our predefined inclusion criteria and were used to extract the full data. Between 2000 and 2015, studies were conducted in 15 out of the 25 countries of the MENA region (Table 1). Algeria, Bahrain, Mauritania, Oman, Palestine, Somalia, Sudan, Syria and UAE lacked studies on NoV. The largest number of studies was reported from Turkey (*n* = 8), followed by Iran (*n* = 6) and Tunisia (*n* = 5). The reported NoV infection rates in MENA countries ranged between 0.82% as reported in Morocco [33] and 36.84% in Tunisia [34]. When applicable, we calculated the median percentage of NoV infection in individual countries. Consequently, we report a 15.13% median percentage of positive NoV in the MENA region based on reported rates across countries (Table 1).

**Table 1:** Norovirus infection in countries of the MENA region between 2000 and 2015

Country	Number of Studies	Minimum Rate	Maximum Rate
Djibouti	1	25.33%	
Egypt	4	13.48%	26.00%
Iran	6	4.14%	32.92%
Iraq	1	30.00%	
Israel	2	9.95%	17.280%
Jordan	1	11.41%	
Kuwait	1	8.23%	
Lebanon	1	6.32%	
Libya	2	15.48%	17.50%
Morocco	2	0.82%	16.12%
Qatar	1	28.47%	
Saudi Arabia	3	3.56%	4.58%
Tunisia	5	8.99%	36.84%
Turkey	8	9.81%	27.70%
Yemen	1	10.35%	
MENA	39	0.82%	36.84%

Based on the World Bank classification, our review includes studies from the following classes of countries: low-income, Yemen; lower-middle income, Djibouti, Egypt, Iran, Iraq, Jordan, Morocco, and Tunisia; upper-middle income, Lebanon, Libya and Turkey and high-income countries, Israel, Kuwait, Qatar and Saudi Arabia. The reported rates of NoV infection ranged between 10 and 37% (Table 1) in lower-middle income and upper-middle income countries. Studies from Israel, Kuwait, Qatar and Saudi Arabia, classified as high-income countries, reported variable rates of 10-17%, 8%, 28%, and 0.4%-5%, respectively (Table 1). In summary, NoV clearly contributes to similar proportion of disease in low, middle- and high-income settings of the MENA region.

The majority of studies on NoV in the MENA region were clinical observational studies aiming at assessing NoV rates mainly among children. Three studies originating from Djibouti, Iran and Qatar reported on NoVs among adults or other non-specified age groups (all ages). Seventeen studies were conducted among children less than 5 years old (15 studies specified age at less than 5 and 2 studies specified age groups at less than 1 and less than 3 years old). While children less than 5 years old constituted the majority of studied target groups, many studies reported on the association of NoV with AGE among children less than other age groups (< 6-18 years). This variability in reporting age makes it difficult to categorize studies based on age.

When looking at the setting, we noted that study participants were recruited from in- or outpatient clinics (Table 2). In summary, 10 studies included inpatients less than 5 years of age (with one study reported on age group less than 3 years). Six other studies were also conducted on in-patients from age groups less than 6, 7, 10, 14 and 17 years old and one on adults (Djibouti) without a specific age category. Two studies from Egypt and a single one from Libya reported on NoV rates among outpatient children. The variability in reporting is complicated by reporting the setting as both in- and outpatient as reported in 13 studies among different age groups. Similarly, the variability in the reported results extends to the study periods during which NoVs were detected from stool of study participants (between 2 months and 7 years) (Table 2).

**Table 2:** Distribution and molecular characteristics of Norovirus in countries of the MENA region (2000-2015)

Country	Population	Participants’ Setting	Study Period (months)	Detection Method	n	Positive Rate	Seasonal Peaks	Predominant Genogroup/Genotype	Article Number
Djibouti	Adults	Inpatients	18	RT-PCR	75	25.34%		GII.14	[55]
Egypt	Children <3	Inpatients	24	RT-PCR	86	25.58%		GII	[56]
	Children <15	Outpatients	12	RT-PCR	500	16.20%			[57]
	Children <5	Outpatients	36	EIA	2112	26.00%	Summer		[58]
	Children <18	In and Outpatients	12	RT-PCR	230	13.48%	Winter	GII.4	[59]
Iran	Infants <1	In and Outpatients	3	IC	82	32.92%			[60]
	Children <7	Inpatients	24	EIA	375	12.53%	Fall		[61]
	Children <5	Inpatients	NM	RT-PCR	2170	4.14%			[62]
	All Ages	Inpatients	12	RT-PCR	293	9.89%		GII.4	[63]
	Children <5	Inpatients	24	Nested RT-PCR	143	6.29%		GII	[64]
	Children <17	Inpatients	24	Nested RT-PCR	47	21.30%		GII.4	[65]
Iraq	Children <5	Inpatients	2	RT-PCR	260	30.00%		GII.4	[66]
Israel	Children <5	In and Outpatients	84	rRT-PCR	673	9.95%		GII.4	[67]
	Children <5	Inpatients	38	rRT-PCR	515	17.28%		GII.4	[68]
Jordan	Children <5	Inpatients	24	rRT-PCR	368	11.41%		GII.3	[69]
Kuwait	Children <5	Inpatients	9	ELISA / RT-PCR	170	5.23%		GII.4	[70]
Lebanon	Children <10	Inpatients	2	EIA / RT-PCR	79	6.32%		GII	[71]
Libya	Children <5	In and Outpatients	12	rRT-PCR	520	17.50%	Summer	GII.4	[72]
	Children <5	Outpatients	8	EIA	239	15.48%	Fall		[39]
Morocco	Children <5	Inpatients	12	Multiplex RT-PCR	122	0.82%	Winter		[33]
	Children <5	Inpatients	12	rRT-PCR	335	16.12%	Summer	GII.4	[73]
Qatar	All Ages	In and Outpatients	6	Multiplex rRT-PCR	288	28.47%			[74]
Saudi Arabia	Children <6	In and Outpatients	12	ELISA	253	3.56%	Fall and Spring		[37]
	Children <5	Inpatients	6	EIA	284	4.58%			[38]
Tunisia	Children <6	Inpatients	18	rRT-PCR	114	36.84%	Winter	GII.3	[34]
	Children <6	Outpatients and Community	12	Multiplex RT-PCR	178	8.99%	Fall and Winter	GII.3	[75]
	Children <13	In and Outpatients	36	RT-PCR	407	9.34%	Winter	GII.3	[76]
	Children <12	In and Outpatients	52	RT-PCR	788	16.24%	Fall and Winter	GII.4	[77]
	Children <12	In and Outpatients	30	RT-PCR	632	17.40%	Winter and Summer	GII.4	[78]
Turkey	Children <17	In and Outpatients	12	Multiplex RT-PCR	240	23.34%			[79]
	Children <16	In and Outpatients	24	IC	1027	10.90%	Spring and Winter		[41]
	Children <5	In and Outpatients	16	RT-PCR	150	10.00%		GII	[80]
	Children <16	Inpatients	12	IC	520	9.81%	Summer		[40]
	Children <6	NM	11	ELISA / RT-PCR	150	22.80%		GII.4	[81]
	Children <14	Inpatients	16	ELISA / RT-PCR / rRT-PCR	238	15.13%		GII.4	[82]
	Children <6	NM	11	Multiplex RT-PCR / EM	144	27.70%		GII.4	[83]
	Children <10	Inpatients	8	RT-PCR	88	17.05%		GII.4	[84]
Yemen	Children <5	In and Outpatients	17	RT-PCR	290	10.35%		GII.4	[85]

### Diagnostics, seasonality and variability

When assessing the diagnostics, the majority of studies (*n* = 30) relied on RT-PCR to detect NoV in stool of study participants. RT-PCR is the most sensitive and specific method to diagnose NoVs. Nevertheless, the use of rapid detection methods (i.e. EIA, ELISA or IC) was reported in 12 studies. Studies relying on molecular assays were able to report on the circulating genotype associated with NoV gastroenteritis (*n* = 25 studies). GII was reported in all the 25 studies with GII.4 predominantly detected in stool of participants as reported by 16 out of the 25 studies (64%). GII.4 circulated in Egypt, Iran, Iraq, Israel, Kuwait, Libya, Morocco, Tunisia, Turkey and Yemen followed by GII.3 (reported in 4 studies, 16.0%) isolated in Jordan and Tunisia.

**Fig. 2:**
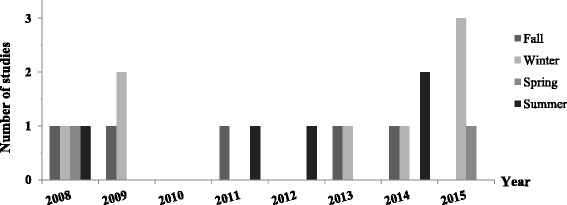
Number of studies reporting seasonal peaks of NoV infection in MENA countries (2000-2015)

A recent systematic review on the global seasonality of NoV infection [35], recognized the latter as a “winter phenomenon” especially in the northern hemisphere. In an attempt to compare regional data from MENA countries to globally reported ones, we extracted data when available on seasonality from studies conducted on NoV between 2000 and 2015. Studies extending for 12 months or more reported on the seasonal peaks of NoV (*n* = 15 studies). When described, NoV infection was detected all year round with detectable winter peaks in Egypt, Morocco, Tunisia and Turkey. However, summer peaks of NoV infections were also reported in Egypt, Libya, Morocco, Tunisia and Turkey. In summary, 5 studies reported detectable peaks in the fall, 8 in the winter, 2 during the spring and 1 during the summer season (Fig. 2). This variability requires further investigation due to the narrow time frame during which hospitalization due to diarrhea was reported and stool tested.

We attempted to address the variability among the studies on NoV from the MENA region in relation to age, settings and diagnostic tool. Studies on participants less than 5 years old reported higher NoV rates than those of older age. This trend was clearly observed in studies from Egypt where NoV infection rates of 26% were reported among children under 5 years old compared to 13.48% and 16% in studies including children less than 18 years (Table 2). A similar trend was observed in Iran where higher rates were observed among infants less than 1 year old (32.92%) compared to the other studies reporting on older children. These results are in agreement with previously reported ones whereby NoV is a significant cause of AGE among children under age of five [19].

Two studies from Israel recruited children of similar age groups and participants’ settings and detected NoV using rRT-PCR. Thus, these studies could be safely considered comparable. The difference in NoV rates between the two studies (9.95% versus 17.28%) could be attributed to the different study periods (7 versus 3 years, respectively); however, we cannot confirm this variability. While two studies from Morocco reported NoV (using RT-PCR) from inpatients less than 5 years old, a wide range of infection rates was reported (i.e. less than 1% vs. 16%) as well as opposite seasonal peaks (winter vs. summer). Using RT-PCR, data reported from Tunisia were from similar settings (in- and out-patients, 4/5 studies), albeit among different age groups. Importantly, NoV rates were the highest among in-patients less than 6 years of age (37% vs. 9-17% among other age groups). The largest number of studies originated from Turkey during the past 15 years. These studies were extremely variable in terms of settings and age groups as shown in Table 2.

When rRT-PCR was used, higher rates of NoV infection were reported compared to those reporting the use of multiplex RT-PCR. This was confirmed in studies from Morocco (16.12% versus 0.82%) and Tunisia (36.84% versus 8.99%). The low rates reported in studies from Saudi Arabia (3.56% and 4.58%) might be an underestimation of NoV-associated gastroenteritis especially since rapid detection methods were used (ELISA and EIA); the latter are less sensitive and specific than RT- PCR [36–38]. Similar findings were detected in Libya where EIA was used [39] and in Turkey where IC was used as detection methods (lowest rates) [40, 41] compared to RT-PCR. We could not assess the heterogeneity in studies from Iraq, Jordan Kuwait, Lebanon, Qatar and Yemen since only one study on NoV was reported in each of these countries.

## Discussion

While the status of NoV-associated AGE is well established in many parts of the world, limited data exist about the contribution of NoV to the burden of diarrheal disease in countries of the MENA region. This prompted us to perform a systematic review providing a summary of the current status of NoV across all age groups and settings in the region. The gathered data suggest that NoV imposes a burden of AGE in the MENA region among children less than 5 years old. The rate of NoV in these studies ranged between 0.82% and 32.9%. These results are compatible with data compiled from studies conducted in Latin America and Africa whereby infection rates ranged between 2.2%-43% [42] and 0.58%-22.0%, [43] respectively. While NoV is associated with AGE among children, recent studies clearly show that other age groups including elderly are at high risk due to the mode of transmission and the severity of symptoms [16]. Only 3 studies on NoV were conducted among all age groups in the MENA region. Consequently, more studies are needed to assess the burden of viral AGE among these groups as well as elderly and immunosuppressed patients at risk of several complications [44, 45]. Moreover, most of the studies included in this systematic review were conducted among in- and outpatients from hospitals. Thus, community studies are needed to assess the community prevalence rates of NoV infection. Importantly and due to living in suboptimal conditions of hygiene and sanitation, community studies assessing NoV infection among displaced populations are also important in conflict areas, many of which are located in the MENA region [46, 47].

Our review clearly shows that NoV GII.4 is predominantly associated with reports of AGE across all age groups in the MENA region (16 studies out of 39; 40%). These results are compatible with data from the Americas, Europe, Asia and Africa [48, 49] whereby GII.4 is reported to be responsible for approximately 55-85% of the gastroenteritis cases worldwide. NoV GII.4 was responsible for a number of pandemics. These pandemics occurred during 1996-1997, 2002, 2004, 2006, 2009, and 2012 [50]. Globally, the GII.4 Sydney strain was associated with outbreaks during the 2012-2013 winter season [51]. This review does not allow us to report on the similarities between the reported NoVs in studies from MENA countries and viral variants circulating or spreading worldwide during the same time periods due to the lack of reporting of sequencing data in the majority of these studies.

When assessing the seasonal pattern of NoV, data from MENA countries clearly show an all year circulation of the virus; however, NoV was mostly detected during winter season as reported by 40% of the included studies followed by fall and summer (25% each). A recent systematic review addressing the global seasonality of NoV infections reported a variable pattern with peaks observed during the winter season; i.e. December -February in the Northern Hemisphere and June - August in the Southern Hemisphere [35]. Importantly, this review used data from regional or national surveillance systems. These systems report on outbreaks from diverse settings including schools, nursing homes, hospitals and others. Countries of the MENA region are geographically located in the Northern Hemisphere where NoV AGE cases peak during the winter season. To the best of our knowledge, NoV is not included in active or passive surveillance systems of MENA countries to help in continuously monitoring incidence/prevalence of NoV associated-AGE. Consequently, we suggest a cautious interpretation of the reported seasonality in studies from MENA countries. Seasonality is impacted by changes in environmental conditions, humidity, temperature cycles, rain patterns and winds affecting NoV transmission [52]. The highly variable data shown in this systematic review, the diverse settings and the short duration of studies limit our ability to clearly identify the factors affecting NoV transmission as previously suggested [35].

Diarrheal diseases are indicators of the development scale of countries. Countries of the MENA region are of variable levels of socioeconomic status as well as sanitation infrastructure suggested to impact the transmission of noroviruses. The availability of and access to safe drinking water and sanitation are key indicators of diarrheal morbidity and mortality and determinants of integral prevention measures to reduce the burden of NoV infections [53, 54]. Our review does not capture data on sanitation and access to clean water.

Our systematic review has a number of limitations. The age groups reported in countries of the MENA region were extremely variable; consequently, the stratification of these target groups was difficult. This is especially important to draw conclusions regarding the gradient of susceptibility among different groups including older individuals. A clear gap exists for data on NoV prevalence in many high-mortality developing countries; consequently we believe that this underrepresentation has clear impact on the assessment of the burden of the disease in the region. Moreover, the comparability between studies is hampered by variability in study design including age groups and methods of detection. While GII.4 was predominantly reported, viral variants in MENA countries could not be deduced in order to assess the role played by pandemic strains in the region. Importantly, our review lacks pooled estimates of NoV prevalence due to the variability and the heterogeneity between studies and settings over time. Consequently, we are not able to generalize these reports to the population level. Further studies are needed where the target group is a close representation of the national population in relation to relevant variables.

## Conclusion

Overall, this review highlights the increasing recognition of NoV as an important causative agent of AGE across all age groups in countries of the MENA region. Further studies are needed to assess the extent of NoV molecular diversity among different age groups. The continuous monitoring of infection among different age groups is important to develop estimates of the regional burden of NoV-gastroenteritis. The introduction of NoV to the panel of surveillance is important to successfully monitor the impact of the virus on the national and regional burden of disease, the diversity of viral variants and seasonal fluctuations. This knowledge is needed to support intervention strategies and to detect new circulating variants possibly associated with underrepresented increased rates of morbidity and mortality in MENA countries.

## Abbreviations

AGE: Acute gastroenteritis
DALY: Disability-adjusted life years
EIA: Enzyme immune assay
ELISA: Enzyme-linked immunosorbent assay
IC: Immunochromatography
LA: Latex agglutination
MENA: Middle East and North Africa
NoV: Norovirus
PRISMA-P: Preferred reporting items for systematic review and meta-analysis protocols
PROSPERO: International prospective register of systematic reviews
rRT-PCR: real time RT-PCR
RT-PCR: Reverse transcriptase polymerase chain reaction
RV: Rotavirus
WHO: World Health Organization
